# Telocytes in the Female Reproductive System: Up-to-Date Knowledge, Challenges and Possible Clinical Applications

**DOI:** 10.3390/life12020267

**Published:** 2022-02-10

**Authors:** Martin Klein, Mária Csöbönyeiová, Ľuboš Danišovič, Lenka Lapides, Ivan Varga

**Affiliations:** 1 Institute of Histology and Embryology, Faculty of Medicine, Comenius University, Sasinkova 4, 811 08 Bratislava, Slovakia; maria.csobonyeiova@fmed.uniba.sk (M.C.); lenka.lapides@gmail.com (L.L.); ivan.varga@fmed.uniba.sk (I.V.); 2 Institute of Medical Biology, Genetics and Clinical Genetics, Faculty of Medicine, Comenius University, Sasinkova 4, 811 08 Bratislava, Slovakia; lubos.danisovic@fmed.uniba.sk; 3 ISCARE, Reproduction Clinic, Gynaecology & Urology, 821 09 Bratislava, Slovakia

**Keywords:** telocytes, female reproductive system, ovaries, uterine tubes, uterus, vagina, placenta, mammary gland

## Abstract

From their initial description in 2005 to this day, telocytes (TCs) have been described in the ovary, uterine tubes, uterus, vagina, mammary gland, and placenta. Their morphological features, immunophenotype, physiological functions, and roles in disease have been thoroughly documented in both animal models and human subjects. TCs, with their extremely long cytoplasmic processes called telopodes, play a pivotal role in the morphological and functional interconnection of all the components of the interstitial compartment, but also with constituents of the parenchyma. Although there is no specific immunohistochemical marker for their identification, the most cited are CD 117, CD 34, platelet-derived growth factor receptor (PDGFR), vimentin, and specific markers typical for the female reproductive system (FRS)—estrogen and progesterone receptors (ER and PR). This immunophenotype provides important clues to their physiological roles. Their main functions include the regulation of hormone-dependent processes, intercellular signaling, immune surveillance, microenvironmental maintenance, and the nursing of stem cells. In a situation where TCs are functionally or morphologically decimated, many disease entities may develop, including premature ovarian failure, endometriosis, ectopic pregnancy, infertility, preeclampsia, or even breast cancer. The common denominator of many of these conditions is that their etiopathogenesis is either partially known or completely obscure. Even though the exact role of TCs in these conditions is yet to be revealed, multiple lines of research indicate that their future clinical application may enrich diagnostic-therapeutic strategies of countless conditions. TCs are also heavily debated in terms of their possible use in regenerative medicine and tissue engineering. Some of the concepts related to TC research are strongly substantiated by experimental data, while others are highly speculative. Only future research endeavors will clearly distinguish dead-end lines of research from genuine contributions to the field.

## 1. Introduction

The female reproductive organs form a highly complex and intricately connected system with many peculiarities unlike any other when we consider other organ systems of the mammalian body. The most astonishing feature of the female reproductive system (FRS) is its capability to induce immune tolerance towards the hemiallogenic embryo. Although this characteristic has been evolutionarily conserved for tens of millions of years, we still do not fully understand it, despite the fact that over the last few decades, the knowledge of the morphology, physiology, and pathophysiology of the FRS has been growing exponentially [1,2]. The FRS’s main task is to ensure the successful development of an embryo/fetus with the ultimate goal of species preservation. In order to fulfill this goal, all the organs belonging to the FRS must work in highly regulated cooperation and synchrony [3]. If any of the components fail to operate properly, various pathological conditions may occur. One of the most significant, which has become a global problem, especially in developed countries, is female-factor infertility [4]. Another clinically important issue is the proneness of some FRS organs to cancerogenesis [5]. The most challenging aspect is that the exact mechanisms by which many of these conditions occur are largely unknown. However, there is one cell population that may be a game-changer in understanding the intricate functioning of the system as a whole—telocytes (TCs) [6].

This paper aims to provide a comprehensive review of TCs in the organs of the FRS, focusing mainly on the past five years, with respect to their physiological roles, potential contribution to the pathogenesis of different FRS-related conditions, and the current understanding of TC roles in regenerative medicine concerning the FRS. In order to do so, we have searched the PubMed/MEDLINE, Scopus, and Web of Science databases to find relevant articles, mainly from the above-mentioned time span, specifically looking for original papers with experimental data on both human subjects and animal models.

## 2. General Information on Telocytes—Morphology and Physiological Functions

The history of TCs can be dated back to the works of Santiago Ramón y Cajal, published at the turn of the 19th and 20th century, who discovered a new cell population of “interstitial neurons” in the gut [7]. A century later, research teams had been studying these cells, now known as interstitial cells of Cajal (ICCs), a designation honoring the Nobel laureate, even though these “interstitial neurons” were, in fact, gut pacemakers, not genuine neurons [8]. In an attempt to find similar cells in other organs, Romanian morphologists under the guidance of prof. Laurentiu M. Popescu discovered a unique yet completely unrecognized cell population. First known as interstitial Cajal-like cells (ICLCs), these cells were later renamed TCs for the sake of simplicity and to emphasize their individuality [9]. 

The shortest and most pertinent definition is that TCs are cells with telopodes. Telopodes represent the most striking morphological feature of these cells—cytoplasmic projections of extraordinary proportions. Their length is surpassed only by that of nerve cell axons [10]. The most thoroughly studied aspect of TCs is their specific morphology. TCs have a small cell body of different shapes. The most commonly described are spindle-shaped, star-shaped, triangular, or piriform TCs [11]. Telopodes, on the other hand, are exquisite in proportions. They can extend from tens to hundreds of micrometers, but are only a fraction of a micrometer wide, making them difficult to study using standard light microscopy. Therefore, the gold standard of TC study is transmission electron microscopy [12]. The length is not the only peculiarity of telopodes. Another important feature of these projections is their moniliform appearance, with alternating thin (podomers) and thick (podoms) segments. Their main function is the formation of homocellular and heterocellular junctions with other components of the stromal compartment, but also functional parenchymal structures such as epithelial cells. In this manner, TCs connect themselves with basically the whole cellular microenvironment of a given organ [13]. This arrangement suggests that telocytes are involved in structural organization, intercellular signaling, microenvironmental maintenance, mechanotransduction, and immune surveillance [14]. Specifically, in the FRS, TCs also serve as hormonal sensors that regulate hormone-dependent physiological and pathological processes [15]. Finally, yet importantly, TCs were also described in close relation to stem cells, which implies their possible importance in tissue regeneration and repair [16]. Apart from direct contact via cell junctions of different types, TCs are also a source of various extracellular vesicles, like exosomes, ectosomes, and multivesicular cargos. These structures contain different molecules that mediate the TC′s influence on its surroundings in a paracrine way [17]. Most of the aforementioned characteristics are universal for TCs in humans, as well as other animal species, irrespective of the particular organ or organ system. Nevertheless, slight differences have been described in the literature. These TC peculiarities, specifically concerning the FRS, are covered in the next sections.

## 3. Morphological and Functional Specifics of FRS Telocytes 

Without overstating, it can be said that TCs have been described in almost every organ one can think of. Except of the FRS, TCs have been discovered in the intestine [18], lungs [19], skeletal muscle [20], skin [21], ureter [22], heart [23], testes [24], prostate [25], eye [26], and even in remarkable locations such as a cow´s teat [27]. TCs in the FRS are quite alike to TCs in other organs, but there are several morphological and functional characteristics that make them distinct. The first unique trait of FRS TCs, in general, is their immunohistochemical profile (details are covered in the next section). For instance, they are positive for estrogen and progesterone nuclear receptors (ER and PR), implying their role in the regulation of hormone-mediated processes occurring in FRS organs. The other significant individuality is that TCs dynamically change their morphology and function based on the current period of the female reproductive cycle, whether it is the menstrual or estrous cycle, and also change noticeably during pregnancy. According to observations by Vannnucchi and Faussone-Pellegrini, such changes may seem to indicate that these are separate populations of TCs, each active in different stages of female reproduction. However, their positivity for both steroid hormone receptors suggests that they are the same, yet morphologically and functionally different [28].

Ovarian TCs are among the least studied compared to other organs of the FRS. Liu et al. studied telocytes in mice ovarian stroma. Using a combination of different methods, including immunofluorescence, immunohistochemistry, flow cytometry, and electron microscopy, the authors identified cells with a small body, long dichotomously branching telopodes with a typical moniliform alternating pattern of podomers and podoms. From the functional perspective, the most probable is their participation in microenvironmental maintenance [29]. Mazzoni et al. studied TCs in the stroma of piscine gonads. In the ovaries, TCs established homo- and heterocellular contacts with all interstitial components, e.g., fibroblasts, theca cells, and blood vessels. The authors hypothesized that TCs might contribute to tissue remodeling of the ovary [30]. Mokhtar also described TCs in fish ovarian stroma making close contacts with immune cells, blood vessels, and atretic follicles, comprising around 8% of the total cellular makeup of the stromal compartment. They were also shown to shed extracellular vesicles in close vicinity to blood capillaries. From the functional perspective, TCs were hypothesized to regulate tissue regeneration during the spawning season [31]. A more recent 2020 paper studying the process of follicular atresia in fish arrived at a conclusion that TCs cooperate with other cell populations, like rodlet cells (special cells present in some epithelia in fish with secretory, sensory, and immune functions), immune cells, and follicular cells in this physiological process by reorganizing the extracellular matrix (ECM) [32].

Tubal TCs, on the other hand, are among the best studied in the FRS. They were first scrutinized shortly after the first description of ICLCs by the Romanian team in 2005. Popescu et al. used a wide spectrum of methods in order to thoroughly examine these cells, but most importantly, they extended the “gold standard” of ultrastructural criteria for their identification, originally derived from the ultrastructural study of ICCs. They established the “platinum standard” that incorporated all typical features of the “characteristic cytoplasmic processes”, not yet known as telopodes, including their length, thickness, branching pattern, and organization. The authors also proposed several hypothetical physiological functions based on their morphological specifics–intercellular signaling, paracrine regulation of their surroundings, pacemaking, and mediation of neurotransmission. Interestingly, the authors also considered the possibility that they can act as further-differentiating progenitor cells or cells capable of phenotypic switch [33]. In a later study, Popescu et al. investigated the topographical representation of TCs in individual histological layers of the uterine tube. Their density was highest in the lamina propria right under the epithelial lining, progressively decreasing towards the more superficial layers of the tubal wall [34]. Eventually, after discovering that they also express ER and PR, Cretoiu et al. discussed that TCs might regulate the hormone-dependent physiology of the uterine tube, e.g., its motility, expanding the palette of probable functions [35]. 

Uterine TCs display several characteristics similar to those found in the uterine tube. They also express ER and PR, implying that TCs serve as hormone sensors and thus regulate hormone-dependent changes associated with the endometrial cycle, pregnancy-related changes, and motility [36,37]. Salama performed original research in female rats divided into four groups based on the reproductive state: immature, adult non-pregnant, adult pregnant, and postpartum rats. The author found TCs in the endometrium and myometrium of all groups, but with quantitative differences. The least numerous population was found in the myometrium and endometrium of immature rats, with a significant increase in non-pregnant adult rats. The pregnant group had a high count of TCs in the endometrium and a low count in the myometrium. The myometrium of the postpartum group contained an abundance of TCs. From these results, it could be inferred that TCs are important during the reproductive period and can adapt according to different functional demands. The high count in the endometrium during pregnancy could indicate that TCs contribute to pregnancy-associated endometrial changes, e.g., decidual reaction. On the other hand, the diminished number of myometrial TCs during pregnancy may prevent pathological contractility and preterm parturition [38]. A recent experimental in vitro study of TCs showed that endometrial TCs influence various physiological characteristics of endometrial stromal cells (ESC). The regulation of ESC proliferation, adhesion, and motility indicate that TCs have a role in the crosstalk between different signaling pathways important for the microenvironmental upkeep of the endometrium [39]. Myometrial TCs were also experimentally demonstrated to express voltage-gated calcium channels, suggesting that TC-mediated calcium fluctuation may determine the proper coordination of myometrial contractility [40]. Jiang et al. studied the immunomodulatory capability of uterine TCs, in terms of their capacity to influence macrophages. TCs were observed to directly activate these innate immune cells through the mitochondrial signaling pathway. Even though the experiment was performed on peritoneal macrophages, the results may suggest that similar processes also occur in the uterine microenvironment [41].

The knowledge of TCs in the vagina is very limited. The only experimental paper studying TCs via immunohistochemical methods was authored by Shafik et al. back in 2005, who concluded that they regulate smooth muscle activity by slow wave initiation [42].

Classifying the placenta as a part of the FRS can be problematic, because it is a temporary or transient organ developing during pregnancy, and on top of that, it is jointly formed by both the maternal and embryonic contribution. Nevertheless, we will describe placental TCs as a part of the FRS. Placental TCs can be discussed from two perspectives. First is their role in early changes that result from the trophoblast-endometrium interplay during embryo implantation, while the second focuses on TCs role in the mature placenta. In the early stages, the endometrium undergoes decidual changes, while at the same time, the immune response has to be adequately regulated in order to mitigate the recognition of the hemiallogenic embryo as non-self by the immune system. The participation of TCs in these processes can be deduced from the results of the already mentioned Jiang et al. study examining TC influence on immune cells [41]. Focusing on the direct observation of TCs in the chorionic villi of the mature placenta, Nizyaeva et al. studied their ultrastructural individualities in human specimens harvested upon delivery between week 36–39 of gestation. The TC population was found to be heterogeneous with several distinct morphological types. Based on their ultrastructural specifics, the authors concluded that placental TCs contribute to trophoblast differentiation and regulation of the growth of chorionic villi. Placental TCs were also observed in close vicinity to Hofbauer cells (special macrophages of the chorionic villi), providing an insight into the potential immunomodulatory activity of placental TCs [43].

In a like manner, TCs in the mammary gland are often discussed separately, since the mammary gland belongs, from the embryological perspective, to skin derivatives. In spite of that, we will discuss the mammary gland TCs within the scope of FRS, considering the fact that it is closely connected to reproduction. The electron microscopic identification of TCs in the mammary gland stroma was carried out by Gherghiceanu et al., who hypothesized that TCs are crucial in three-dimensional (3-D) organization and functional integration of stromal components of the mammary gland [44]. Petre et al. found TCs in stem cell niches of the mammary gland, indicating that they might be important in morphological and functional changes related to early development, and pregnancy-associated growth and differentiation [45]. In a 2020 original paper, Sanches et al. examined the mammary gland of the Mongolian gerbil with a goal to perform an ultrastructural and immunohistochemical study of TCs during lactation and post-lactational involution. The authors observed that their function was closely associated with matrix metalloproteinase 9 (MMP-9) and vascular endothelial growth factor (VEGF), suggesting their capacity for ECM remodeling and angiogenesis regulation, respectively [46]. 

## 4. Immunophenotype of FRS Telocytes

Besides electron microscopic features, immunophenotypic traits are the most cited TC characteristics by which they can be distinguished. Unfortunately, as of today, no research endeavors have been successful in determining a unique and specific marker that would unambiguously differentiate TC from other populations of interstitial cells. Nevertheless, there are several immunohistochemical markers that can be considered reliable in TC identification. The most suitable approach is the combination of markers as a part of double immunohistochemistry, immunocytochemistry, immunofluorescence. These can also be used in tandem with other methods of TC detection. However, it is extremely important to note that there are organ-to-organ differences in the expression of these markers, which makes the straightforward detection of TCs even more challenging than the fact that there is no TC-specific marker.

The handful of studies that we have at our disposal showed that ovarian TCs express CD 34, platelet-derived growth factor receptor (PDGFR) α/β, vimentin [29], MMP-2 and MMP-9 [30], desmin, CD 117 (c-kit), and S-100 protein [31].

Regarding the immunophenotype of TCs in the uterine tube, Cretoiu published a comprehensive review on different aspects of tubal TCs, including the most typical markers detectable via immunohistochemistry or immunofluorescence. A marker that displayed the strongest positivity was CD 117, followed by CD 34. The former was located mostly on the cell body, while the latter was found predominantly on telopodes. S-100 positivity was also demonstrated with variations found in different histological layers of the uterine tube. The expression of other markers, namely NK-1, nestin, and vimentin, was either weak or inconsistent. Finally, yet importantly, tubal TCs expressed ER and PR, but also atypical markers caveolin-1 and caveolin-2 [34,47].

Similar to those in the uterine tubes, uterine TCs are also thoroughly studied from various angles, including the expression of immunohistochemical markers. There is a substantial overlap with other organs of the FRS, especially the uterine tube, but as always, previous experimental works found several organ-specific markers. The immunophenotypic details of the uterine TCs were elucidated only one year after their initial discovery, indicating that uterine TCs are among the best documented overall, not only in the FRS. They express CD 117, CD 34, vimentin [48], ER, and PR [36]. Our own previous experiments also demonstrated that uterine TCs are CD 117 positive in different histological layers of both the uterine body and cervix, with the strongest positivity found in the myometrium [49]. Apart from these typical markers, Hatta et al. and Rosenbaum et al. demonstrated that uterine TCs are positive for connexin 43 and SK3 channels, respectively [47,50,51]. T-type Ca2+ channels and PDGFR α are markers that can also be found in uterine TCs. To mitigate the no-single-specific-marker problem, the best course of action is the marker combination in terms of double immunohistochemistry. The most reliable merger seems to be CD 34/PDGFR α [52]. 

CD 117 positive TCs in the vagina were described by Shafik et al., though they referred to these cells ambiguously as either pacemaking ICCs or ICLCs [42]. 

In 2007, Suciu et al. used a multimodal approach to studying placental TCs, including immunofluorescence and cell cultivation. The authors described TCs (ICLCs) positive for CD 117, vimentin, α- smooth muscle actin (SMA), and caveolin-1 [53]. Several years later, in 2010, the same research group performed a more detailed analysis of TCs in the human term placenta. They managed to identify additional markers, namely CD 34, VEGF, inducible nitric oxide synthase (iNOS), CD 44, nestin, S-100 protein, neuron-specific enolase (NSE), and desmin [54].

A 2013 study by Mou et al. revealed that mammary gland TCs express typical markers, such as CD 117, vimentin, and CD 34 [55]. A more recent 2019 study by El-Tahawy et al. confirmed that CD 117 and CD 34 could be considered reliable for TC identification in the mammary gland [56].

## 5. Pathology of FRS Telocytes

Liu et al. established an animal model of cyclophosphamide-induced premature ovarian failure to study the potential role of ovarian TCs in its pathogenesis. The authors provided ultrastructural and immunohistochemical evidence of TC absence in the experimental group, suggesting that ovarian TCs might be important for the maintenance of the ovarian interstitial compartment. After TC loss and ensuing microenvironmental deregulation, premature ovarian failure may develop [29]. Like in most other cases, when a research group finds that TCs are absent in some pathological state, it is hard to distinguish whether the TC loss is a mere consequence of a noxious influence; thus, their absence is only a correlation, or the loss of TCs from a different reason is then causally related to the development of a given disease. 

There is a great abundance of publications dealing with the potential roles of tubal TCs in various pathological conditions. Based on their ultrastructural, functional, and immunophenotypic traits, tubal TCs have been considered in the pathogenesis of such conditions as tubal endometriosis, tubal infertility, ciliary dyskinesia, or even ectopic pregnancy. Aleksandrovych et al. conducted research on tubal specimens from human subjects diagnosed with uterine leiomyomas (fibroids). This recent study brought several surprising findings that may provide the necessary momentum to push the research of tubal TCs further. TCs were found to be positive for ER, PR, and SK3, secreted VEGF, and interacted with protein gene product 9.5 (PGP 9.5), iNOS, and choline acetyltransferase (ChAT)-positive nerve fibers. Taken together, the authors concluded that tubal TCs are involved in muscle contractility, angiogenesis, and overall maintenance of the local microenvironment. Such a broad spectrum of functional involvement indicates that when tubal TCs are damaged, various pathological changes may emerge. The most significant is tubal-factor infertility. A completely unique and previously unrecognized finding was that the number of tubal TCs in patients with uterine fibroids increased, possibly as a compensatory mechanism. Unfortunately, a more detailed explanation of this phenomenon was not provided [57]. An entirely new 2022 study on tubal TCs came up with another discovery, unlike any other to this date. Karasu et al. studied human specimens from patients diagnosed with ectopic pregnancy. They observed that the experimental group had an increased number of TCs in the muscle layer and serosa. The authors concluded that this increase might paradoxically hinder tubal motility, predisposing a patient to ectopic pregnancy [58]. The most peculiar about these results is the fact that in the vast majority of previously published papers, a TC-associated disease was discussed in terms of the loss of TCs (i.e., quantitative reduction), not the other way around. This indicates that a delicate balance is of the utmost importance for TCs to function properly in any given organ.

One of the most thoroughly researched conditions associated with a disturbance of uterine TCs is the development of uterine leiomyomas (fibroids). Leiomyomas are non-malignant smooth muscle neoplasms, that are, despite their non-invasiveness, related to clinically relevant morbidity and fertility problems [59]. The role of uterine TCs in the pathogenesis of uterine leiomyomas has been studied from different aspects. The main rationale stems from the role of TCs as hormone sensors, a function implicated by their above-mentioned positivity for ER and PR. It is known that uterine leiomyomas are hormone-sensitive tumors, so in the situation of quantitative or qualitative alteration of uterine TCs, this loss of regulatory capacity in the myometrium can possibly contribute to their development. Aleksandrovych et al. provided immunohistochemical evidence of diminished count of uterine TCs in leiomyomas [60]. The results of our own original paper on the topic were even more striking—we found no TCs whatsoever in the leiomyoma samples compared to healthy myometrium [61]. We provided two more hypotheses on how TC loss might be related to the pathogenesis of uterine leiomyomas. Based on the previously published role of TCs in the tumorigenesis of extragastrointestinal stromal tumors (eGISTs) [62], uterine TCs might also serve as cells-of-origin of uterine leiomyomas. The other hypothesis was based on the known involvement of TCs in angiogenesis and the known role of hypoxia in the pathogenesis of leiomyomas [63]. This relationship was further investigated and underscored in a more recent 2019 experimental study. In the case of TC absence or malfunction, the limited angiogenic capacity makes the organ more prone to hypoxia, which can, in combination with other factors, lead to leiomyoma formation [64]. Uterine TCs were also found to be directly associated with infertility and early pregnancy loss. These connections originated from in vitro experiments. Chi et al. established a co-culture of TC-conditioned media with peritoneal macrophages. The results indicated that TCs are active participators in local immune regulation. Since the immune system plays a huge role in normal pregnancy, TC disturbance can be the yet unrecognized factor that could potentially elucidate the pathogenesis of these ill-understood conditions [65]. The intensity of interest among scholars to study TC involvement in disease development can be documented by experiments studying rather outlandish associations. Skowron et al. established an animal model of anorexia nervosa to study pathological changes in the reproductive organs resulting from this eating disorder, that is at the same time a serious mental health problem. Within the experimental methodology, the authors also focused on the immunohistochemical identification of TCs. Despite the results, which showed no difference in TC distribution between the experimental and control group [66], these research endeavors document a very interesting trend. The study of TC alteration as the causative factor in disease pathogenesis or the reverse situation when a primary disease can negatively influence resident TCs (leading to additional problems resulting from their loss) can be considered a hot topic in morphological sciences.

To the best of our knowledge, there are no experimental data indicating the involvement of TCs in any vaginal pathology.

The knowledge on placental TCs partially overlaps with that of uterine TCs, since the placenta is formed by the interplay of both maternal and embryonic tissues. The most researched pathological condition concerning placental TCs is preeclampsia. This serious pregnancy-associated pathology typically results from defective placentation, abnormal spiral artery remodeling, placental insufficiency, and endothelial dysfunction. All these interconnected pathological processes eventually lead to ischemic-hypoxic damage. If left untreated, it can lead to life-threatening complications [67]. Placental TCs were located in a strategic position in the chorionic villi next to fetal blood vessels and myofibroblasts. The placenta, as a non-innervated organ, has to use different mechanisms for regulation and signal transduction compared to innervated organs. TCs might be responsible for those signal transduction mechanisms necessary for a normal blood flow in fetal blood vessels and normal motility of the chorionic villi required for a proper metabolic exchange through the placental barrier [68]. If some of these mechanisms are not functioning properly due to TC loss, the affected patients might be at a higher risk of developing preeclampsia [69,70]. It is possible that TCs are important not only in the mature placenta but also in the earliest stages of its development right upon the blastocyst implantation. As the trophoblast invades the endometrium, this layer of the uterine wall undergoes decidual changes that include the differentiation of stromal fibroblasts into decidual cells. The role of TCs in this process can be inferred from an experimental co-cultivation of TCs with stromal cells obtained from the endometrium. This study by Tang et al. showed that TCs influenced various physiological activities of these stromal cells, namely their adhesive, proliferative, and migratory capacities [39]. These results underline a possible involvement of placental TCs in infertility and early pregnancy loss, as discussed in the context of uterine TCs. 

One of the most serious pathologies of the mammary gland is undoubtedly breast cancer. This malignant tumor is the most common type of cancer occurring in women worldwide [71]. Mou et al. investigated the mechanisms of interaction between TCs, various other cells of the mammary gland stroma, and EMT 6 murine mammary carcinoma cell line. This in vitro experiment showed that TCs and other stromal cells were involved in nest formation, proliferation, and apoptosis inhibition. In summary, the authors were able to reconstruct breast cancer in vitro [55]. Recently, Díaz-Flores et al. found experimental support for a hypothesis that TCs might be a source of cancer-associated fibroblasts in patients with invasive lobular carcinoma of the breast [72]. Such a finding is of high clinical importance, since cancer-associated fibroblasts regulate tumor development, metastasizing, and play a part in resistance to therapy [73]. These results are indicative of the possibility that therapeutic targeting of TCs might be a component of future complex state-of-the-art treatment approaches to this serious oncological diagnosis.

It can be summarized that the relationship between quantitative and/or qualitative alteration of TCs in the etiopathogenesis of a diverse spectrum of pathological conditions has been a staple motif in almost the whole bulk of publications on TCs published to this day. Some research teams, e.g., Yonghong et al., went so far as to associate TCs with such speculative concepts, like meridians in traditional Chinese medicine [74]. Regardless of a particular organ, or animal species, TCs have been discussed in the development of so many different pathological conditions that a considerable proportion of researchers have been frowning upon a notion that a singular cell population can have such an impact [75]. Nevertheless, we think that if the causal relationship is firmly established, an umbrella term for these conditions will be convenient. We already proposed one—telocytopathies [75]. 

**Figure 1:**
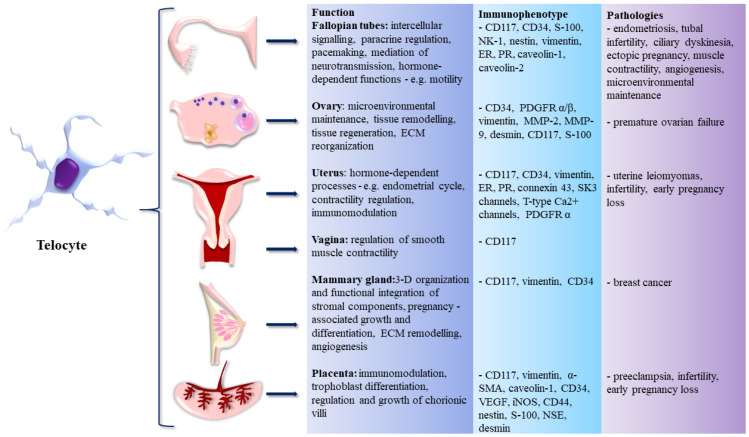
Telocytes (TCs) in the female reproductive system (FRS)—organs of occurrence, immunophenotype, functions, and role in pathogenesis of assorted diseases.

All the sections above are summarized in Figure 1, depicting the occurrence of TCs within the FRS, their immunophenotype, functions, and roles in disease.

## 6. Telocytes and FRS Regeneration

Regenerative medicine of the female reproductive organs is a highly relevant and up-to-date area of research. There are many diagnoses, as well as physiological processes in which the understanding of the mechanisms of tissue regeneration and repair is of paramount importance. Two examples for all are the intricate changes that occur during the menstrual cycle and pregnancy. Over the span of the reproductive period of a woman, uterine endometrium is subjected to around 400 (depending on parity) menstrual cycles that are characterized by a sophisticated coordination of cell proliferation, differentiation, angiogenesis, and subsequent disintegration, and sloughing off. Even more astonishing changes occur in pregnancy, when the weight of the uterus increases tenfold by the processes of hypertrophy and hyperplasia [76]. Discussing the significance of tissue and organ regeneration in a pathological context, there is a rare yet severe congenital anomaly that symbolizes the great challenges that regenerative medicine and tissue engineering of the FRS have to face. It is a condition referred to as Mayer-Rokitansky-Küster-Hauser syndrome, characterized by congenital agenesis of the uterine tubes, uterus, and upper two-thirds of the vagina [77]. While on the one hand, the understanding of regenerative processes and their clinical applications can help with the management of conditions such as endometriosis or infertility, a whole other level is tissue engineering of full-sized organs that can have a profound impact on the lives of many women born with serious congenital defects. However, the latter is way more challenging to accomplish.

Soon after the morphological and functional characterization of TCs, it has become evident that their role in tissue regeneration and repair will be a common denominator in most organs. Concerning uterine TCs, Campeanu et al. experimentally demonstrated that TCs from pregnant and non-pregnant uteri react differently to low-level-laser stimulation (LLLS). Upon LLLS, TCs from pregnant uteri reacted more intensely in terms of dynamic changes in the length, branching, and overall plasticity of telopodes. These results provided conclusive evidence that telopodes are sensitive to physical stimulation, which may open new possibilities in TC-associated regeneration [78]. Apart from the FRS, the regenerative potential of TCs has been previously studied in the heart [79], respiratory system [13], skeletal muscle [80], skin [81], nervous system [82], sensory organs [83], liver [84], and urinary system [85]. Fabricated synthetic scaffolds, decellularized biological scaffolds, 3-D printing technologies, and induced pluripotent stem cells are all at the forefront of clinical applications. Despite the fact that these approaches to regenerative medicine and tissue engineering have been expanding enormously over the last years, there are still many issues that need to be addressed, e.g., in vivo survival of transplanted stem cells, proper restoration of the tissue microarchitecture, revascularization, and most importantly, all of it has to be applicable at the human scale [86]. When we consider the presence of TCs in stem cell niches of various organs, their role in angiogenesis, intercellular signaling, and overall microenvironmental maintenance, it is reasonable to infer that multiple problems associated with successful tissue regeneration could be solved if TCs were put into play. 

## 7. Conclusions and Future Perspectives

A lot has been discovered about TCs in the female reproductive organs over the 16 years that have passed since their initial description. From their peculiar morphology and function, to their roles in various diseases, to their potential in tissue regeneration, TCs contributed largely to many fields of biomedical sciences, including gynecology, obstetrics, and gynecological oncology. There are still many under-researched areas where TCs can provide additional insights into normal and pathological structure and function. The great potential of TCs lies in their ability to broaden the understanding of many diseases, which are now defined as idiopathic (without known cause), and bring novelties to diagnostic-therapeutic management of a wide array of pathological conditions. Finally, yet importantly, their contribution to regenerative medicine and tissue engineering is up-and-coming. 

Some of the concepts related to TC research are strongly substantiated by experimental data, while others are highly speculative. Only future research endeavors will clearly distinguish dead-end lines of research from genuine contributions to the field [87].

## Abbreviations

ChAT	choline acetyltransferase
ESCs	endometrial stromal cells
ER	estrogen receptor
ECM	extracellular matrix
eGISTs	extragastrointestinal stromal tumors
FRS	female reproductive system
iNOS	inducible nitric oxide synthase
ICLCs	interstitial Cajal-like cells
ICCs	interstitial cells of Cajal
LLLS	low-level-laser stimulation
MMP	matrix metalloproteinase
NSE	neuron-specific enolase
PDGFR	platelet-derived growth factor receptor
PR	progesterone receptor
PGP 9.5	protein gene product 9.5
SMA	smooth muscle actin
TCs	telocytes
3-D	three-dimensional
VEGF	vascular endothelial growth factor

## References

[1] Clark G.F., Schust D.J. (2013). Manifestations of immune tolerance in the human female reproductive tract. Front. Immunol.

[2] Bates G.W., Bowling M. (2013). Physiology of the female reproductive axis. Periodontol. 2000.

[3] Schauer C., Tong T., Petitjean H., Blum T., Peron S., Mai O., Schmitz F., Boehm U., Leinders-Zufall T. (2015). Hypothalamic gonadotropin-releasing hormone (GnRH) receptor neurons fire in synchrony with the female reproductive cycle. J. Neurophysiol..

[4] Ledger W.L. (2009). Demographics of infertility. Reprod. Biomed. Online.

[5] Weiderpass E., Labrèche F. (2012). Malignant tumors of the female reproductive system. Saf. Health Work.

[6] Janas P., Kucybała I., Radoń-Pokracka M., Huras H. (2018). Telocytes in the female reproductive system: An overview of up-to-date knowledge. Adv. Clin. Exp. Med..

[7] Junquera C., Martínez-Ciriano C., Castiella T., Serrano P., Azanza M.J., Junquera S.R. (2007). Immunohistochemical and ultrastructural characteristics of interstitial cells of Cajal in the rabbit duodenum. Presence of a single cilium. J. Cell Mol. Med..

[8] Faussone-Pellegrini M.S., Pantalone D., Cortesini C. (1990). Smooth muscle cells, interstitial cells of Cajal and myenteric plexus interrelationships in the human colon. Acta Anat..

[9] Popescu L.M., Faussone-Pellegrini M.S. (2010). TELOCYTES—A case of serendipity: The winding way from Interstitial Cells of Cajal (ICC), via Interstitial Cajal-Like Cells (ICLC) to TELOCYTES. J. Cell Mol. Med..

[10] Cretoiu S.M., Popescu L.M. (2014). Telocytes revisited. Biomol. Concepts.

[11] Kucybala I., Janas P., Ciuk S., Cholopiak W., Klimek-Piotrowska W., Holda M.K. (2017). A comprehensive guide to telocytes and their great potential in cardiovascular system. Bratisl. Lek. Listy.

[12] Cretoiu D., Radu B.M., Banciu A., Banciu D.D., Cretoiu S.M. (2017). Telocytes heterogeneity: From cellular morphology to functional evidence. Semin. Cell Dev. Biol..

[13] Popescu L.M., Gherghiceanu M., Suciu L.C., Manole C.G., Hinescu M.E. (2011). Telocytes and putative stem cells in the lungs: Electron microscopy, electron tomography and laser scanning microscopy. Cell Tissue Res..

[14] Kondo A., Kaestner K.H. (2019). Emerging diverse roles of telocytes. Development.

[15] Urban L., Miko M., Kajanova M., Bozikova S., Mrazova H., Varga I. (2016). Telocytes (interstitial Cajal-like cells) in human Fallopian tubes. Bratisl. Lek. Listy.

[16] Bei Y., Wang F., Yang C., Xiao J. (2015). Telocytes in regenerative medicine. J. Cell. Mol. Med..

[17] Cretoiu D., Xu J., Xiao J., Cretoiu S.M. (2016). Telocytes and Their Extracellular Vesicles-Evidence and Hypotheses. Int. J. Mol. Sci..

[18] Verdile N., Pasquariello R., Cardinaletti G., Tibaldi E., Brevini T.A.L., Gandolfi F. (2021). Telocytes: Active Players in the Rainbow Trout (Oncorhynchus mykiss) Intestinal Stem-Cell Niche. Animals.

[19] Tang L., Song D., Qi R., Zhu B., Wang X. (2022). Roles of pulmonary telocytes in airway epithelia to benefit experimental acute lung injury through production of telocyte-driven mediators and exosomes. Cell Biol. Toxicol..

[20] Ravalli S., Federico C., Lauretta G., Saccone S., Pricoco E., Roggio F., Di Rosa M., Maugeri G., Musumeci G. (2021). Morphological Evidence of Telocytes in Skeletal Muscle Interstitium of Exercised and Sedentary Rodents. Biomedicines.

[21] Chen X., Zeng J., Huang Y., Gong M., Ye Y., Zhao H., Chen Z., Zhang H. (2021). Telocytes and their structural relationships with surrounding cell types in the skin of silky fowl by immunohistochemistrical, transmission electron microscopical and morphometric analysis. Poult Sci.

[22] Wishahi M., Hafiz E., Wishahy A.M.K., Badawy M. (2021). Telocytes, c-Kit positive cells, Smooth muscles, and collagen in the ureter of pediatric patients with congenital primary obstructive megaureter: Elucidation of etiopathology. Ultrastruct. Pathol..

[23] Sukhacheva T.V., Nizyaeva N.V., Samsonova M.V., Cherniaev A.L., Burov A.A., Iurova M.V., Shchegolev A.I., Serov R.A., Sukhikh G.T. (2021). Morpho-functional changes of cardiac telocytes in isolated atrial amyloidosis in patients with atrial fibrillation. Sci. Rep..

[24] Gomes V.L.A., Braz J., Martins G.M., Clebis N.K., Oliveira M.F., Morais D.B., Moura C.E.B. (2021). Identification of telocytes in dystrophic mice testis. Einstein (Sao Paulo).

[25] Felisbino S.L., Sanches B.D.A., Delella F.K., Scarano W.R., Dos Santos F.C.A., Vilamaior P.S.L., Taboga S.R., Justulin L.A. (2019). Prostate telocytes change their phenotype in response to castration or testosterone replacement. Sci. Rep..

[26] Nicolescu M.I., Rusu M.C., Voinea L.M., Vrapciu A.D., Bâră R.I. (2020). Lymphatic lacunae of the human eye conjunctiva embedded within a stroma containing CD34(+) telocytes. J. Cell. Mol. Med..

[27] Wagener M.G., Leonhard-Marek S., Häger J.D., Pfarrer C. (2018). CD117- and vimentin-positive telocytes in the bovine teat sphincter. Anat. Histol. Embryol..

[28] Vannucchi M.G., Faussone-Pellegrini M.S. (2016). The Telocyte Subtypes. Adv. Exp. Med. Biol..

[29] Liu T., Wang S., Li Q., Huang Y., Chen C., Zheng J. (2016). Telocytes as potential targets in a cyclophosphamide-induced animal model of premature ovarian failure. Mol. Med. Rep..

[30] Mazzoni T.S., Viadanna R.R., Quagio-Grassiotto I. (2019). Presence, localization and morphology of TELOCYTES in developmental gonads of fishes. J. Morphol..

[31] Mokhtar D.M. (2019). Characterization of the fish ovarian stroma during the spawning season: Cytochemical, immunohistochemical and ultrastructural studies. Fish. Shellfish. Immunol..

[32] Mokhtar D.M., Hussein M.M. (2020). Microanalysis of Fish Ovarian Follicular Atresia: A Possible Synergic Action of Somatic and Immune Cells. Microsc. Microanal..

[33] Popescu L.M., Ciontea S.M., Cretoiu D., Hinescu M.E., Radu E., Ionescu N., Ceausu M., Gherghiceanu M., Braga R.I., Vasilescu F. (2005). Novel type of interstitial cell (Cajal-like) in human fallopian tube. J. Cell. Mol. Med..

[34] Popescu L.M., Ciontea S.M., Cretoiu D. (2007). Interstitial Cajal-like cells in human uterus and fallopian tube. Ann. N. Y. Acad. Sci..

[35] Cretoiu S.M., Cretoiu D., Suciu L., Popescu L.M. (2009). Interstitial Cajal-like cells of human Fallopian tube express estrogen and progesterone receptors. J. Mol. Histol..

[36] Cretoiu D., Ciontea S.M., Popescu L.M., Ceafalan L., Ardeleanu C. (2006). Interstitial Cajal-like cells (ICLC) as steroid hormone sensors in human myometrium: Immunocytochemical approach. J. Cell. Mol. Med..

[37] Roatesi I., Radu B.M., Cretoiu D., Cretoiu S.M. (2015). Uterine Telocytes: A Review of Current Knowledge. Biol. Reprod..

[38] Salama N.M. (2013). Immunohistochemical characterization of telocytes in ratuterus in different reproductive states. Egypt. J. Histol..

[39] Tang X.L., Zhang F.L., Jiang X.J., Yang X.J. (2019). Telocytes enhanced the proliferation, adhesion and motility of endometrial stromal cells as mediated by the ERK pathway in vitro. Am. J. Transl. Res..

[40] Banciu A., Banciu D.D., Mustaciosu C.C., Radu M., Cretoiu D., Xiao J., Cretoiu S.M., Suciu N., Radu B.M. (2018). Beta-Estradiol Regulates Voltage-Gated Calcium Channels and Estrogen Receptors in Telocytes from Human Myometrium. Int. J. Mol. Sci..

[41] Jiang X.J., Cretoiu D., Shen Z.J., Yang X.J. (2018). An in vitro investigation of telocytes-educated macrophages: Morphology, heterocellular junctions, apoptosis and invasion analysis. J. Transl. Med..

[42] Shafik A., El-Sibai O., Shafik I., Shafik A.A. (2005). Immunohistochemical identification of the pacemaker cajal cells in the normal human vagina. Arch. Gynecol. Obstet..

[43] Nizyaeva N.V., Sukhacheva T.V., Kulikova G.V., Nagovitsyna M.N., Poltavtseva R.A., Kan N.E., Tyutyunnik V.L., Pavlovich S.V., Serov R.A., Shchyogolev A.I. (2017). Ultrastructural Characteristics of Placental Telocytes. Bull. Exp. Biol. Med..

[44] Gherghiceanu M., Popescu L.M. (2005). Interstitial Cajal-like cells (ICLC) in human resting mammary gland stroma. Transmission electron microscope (TEM) identification. J. Cell. Mol. Med..

[45] Petre N., Rusu M.C., Pop F., Jianu A.M. (2016). Telocytes of the mammary gland stroma. Folia Morphol. (Warsz).

[46] Sanches B.D.A., Leonel E.C.R., Maldarine J.S., Tamarindo G.H., Barquilha C.N., Felisbino S.L., Goés R.M., Vilamaior P.S.L., Taboga S.R. (2020). Telocytes are associated with tissue remodeling and angiogenesis during the postlactational involution of the mammary gland in gerbils. Cell Biol. Int..

[47] Cretoiu S.M. (2016). Immunohistochemistry of Telocytes in the Uterus and Fallopian Tubes. Adv. Exp. Med. Biol..

[48] Ciontea S.M., Radu E., Regalia T., Ceafalan L., Cretoiu D., Gherghiceanu M., Braga R.I., Malincenco M., Zagrean L., Hinescu M.E. (2005). C-kit immunopositive interstitial cells (Cajal-type) in human myometrium. J. Cell. Mol. Med..

[49] Klein M., Urban L., Deckov I., Danisovic L., Polak S., Danihel L., Varga I. (2017). Distribution of telocytes in the corpus and cervix of human uterus: An immunohistochemical study. Biologia.

[50] Hatta K., Huang M.L., Weisel R.D., Li R.K. (2012). Culture of rat endometrial telocytes. J. Cell. Mol. Med..

[51] Rosenbaum S.T., Svalø J., Nielsen K., Larsen T., Jørgensen J.C., Bouchelouche P. (2012). Immunolocalization and expression of small-conductance calcium-activated potassium channels in human myometrium. J. Cell. Mol. Med..

[52] Cretoiu S.M., Radu B.M., Banciu A., Banciu D.D., Cretoiu D., Ceafalan L.C., Popescu L.M. (2015). Isolated human uterine telocytes: Immunocytochemistry and electrophysiology of T-type calcium channels. Histochem. Cell Biol..

[53] Suciu L., Popescu L.M., Gherghiceanu M. (2007). Human placenta: De visu demonstration of interstitial Cajal-like cells. J. Cell. Mol. Med..

[54] Suciu L., Popescu L.M., Gherghiceanu M., Regalia T., Nicolescu M.I., Hinescu M.E., Faussone-Pellegrini M.S. (2010). Telocytes in human term placenta: Morphology and phenotype. Cells Tissues Organs.

[55] Mou Y., Wang Y., Li J., Lü S., Duan C., Du Z., Yang G., Chen W., Zhao S., Zhou J. (2013). Immunohistochemical characterization and functional identification of mammary gland telocytes in the self-assembly of reconstituted breast cancer tissue in vitro. J. Cell. Mol. Med..

[56] El-Tahawy N.F.G., Rifaai R.A. (2019). Immunohistochemical and ultrastructural evidence for telocytes in the different physiological stages of the female rat mammary gland. Life Sci..

[57] Aleksandrovych V., Wrona A., Bereza T., Pityński K., Gil K. (2021). Oviductal Telocytes in Patients with Uterine Myoma. Biomedicines.

[58] Karasu Y., Önal D., Zırh S., Yersal N., Korkmaz H., Üstün Y., Müftüoğlu S., Pehlivanoğlu B. (2022). Role of telocytes in the pathogenesis of ectopic pregnancy. Eur. Rev. Med. Pharmacol. Sci..

[59] Stewart E.A., Cookson C.L., Gandolfo R.A., Schulze-Rath R. (2017). Epidemiology of uterine fibroids: A systematic review. BJOG.

[60] Aleksandrovych V., Białas M., Pasternak A., Bereza T., Sajewicz M., Walocha J., Gil K. (2018). Identification of uterine telocytes and their architecture in leiomyoma. Folia Med. Cracov..

[61] Varga I., Klein M., Urban L., Danihel L., Polak S., Danihel L. (2018). Recently discovered interstitial cells “telocytes” as players in the pathogenesis of uterine leiomyomas. Med. Hypotheses.

[62] Padhi S., Nayak H.K. (2016). Primary Extragastrointestinal Stromal Tumours in the Hepatobiliary Tree and Telocytes. Adv. Exp. Med. Biol..

[63] Fletcher N.M., Saed M.G., Abu-Soud H.M., Al-Hendy A., Diamond M.P., Saed G.M. (2013). Uterine fibroids are characterized by an impaired antioxidant cellular system: Potential role of hypoxia in the pathophysiology of uterine fibroids. J. Assist. Reprod. Genet..

[64] Aleksandrovych V., Bereza T., Ulatowska-Białas M., Pasternak A., Walocha J.A., Pityński K., Gil K. (2019). Identification of PDGFRα + cells in uterine fibroids—Link between angiogenesis and uterine telocytes. Arch. Med. Sci..

[65] Chi C., Jiang X.J., Su L., Shen Z.J., Yang X.J. (2015). In vitro morphology, viability and cytokine secretion of uterine telocyte-activated mouse peritoneal macrophages. J. Cell. Mol. Med..

[66] Skowron K., Aleksandrovych V., Kurnik-Łucka M., Stach P., Baranowska A., Skowron B., Gil K. (2018). Aberrations in the female reproductive organs and a role of telocytes in a rat model of anorexia nervosa. Folia Med. Cracov..

[67] Nirupama R., Divyashree S., Janhavi P., Muthukumar S.P., Ravindra P.V. (2021). Preeclampsia: Pathophysiology and management. J. Gynecol. Obstet. Hum. Reprod..

[68] Bosco C.B., Díaz E.G., Gutierrez R.R., González J.M., Parra-Cordero M., Rodrigo R.S., Barja P.Y. (2016). Placental Hypoxia Developed During Preeclampsia Induces Telocytes Apoptosis in Chorionic Villi Affecting The Maternal-Fetus Metabolic Exchange. Curr. Stem Cell Res. Ther..

[69] Bosco C., Díaz E., Gutiérrez R., González J., Parra-Cordero M., Rodrigo R., Barja P. (2015). A putative role for telocytes in placental barrier impairment during preeclampsia. Med. Hypotheses.

[70] Abu-Dief E.E., Elsayed H.M., Atia E.W., Abdel-Rahman M., Fawzy M. (2021). Modulation of Telocytes in Women with Preeclampsia: A Prospective Comparative Study. J. Microsc. Ultrastruct..

[71] Momenimovahed Z., Salehiniya H. (2019). Epidemiological characteristics of and risk factors for breast cancer in the world. Breast Cancer (Dove Med. Press).

[72] Díaz-Flores L., Gutiérrez R., González-Gómez M., García M.P., Díaz-Flores L., Carrasco J.L., Martín-Vasallo P. (2021). CD34+ Stromal Cells/Telocytes as a Source of Cancer-Associated Fibroblasts (CAFs) in Invasive Lobular Carcinoma of the Breast. Int. J. Mol. Sci..

[73] Ping Q., Yan R., Cheng X., Wang W., Zhong Y., Hou Z., Shi Y., Wang C., Li R. (2021). Cancer-associated fibroblasts: Overview, progress, challenges, and directions. Cancer Gene Ther..

[74] Yonghong S., Ruizhi W., Yue Z., Xuebing B., Tarique I., Chunhua L., Ping Y., Qiusheng C. (2020). Telocytes in Different Organs of Vertebrates: Potential Essence Cells of the Meridian in Chinese Traditional Medicine. Microsc. Microanal..

[75] Varga I., Polák Š., Kyselovič J., Kachlík D., Danišovič Ľ., Klein M. (2019). Recently Discovered Interstitial Cell Population of Telocytes: Distinguishing Facts from Fiction Regarding Their Role in the Pathogenesis of Diverse Diseases Called “Telocytopathies”. Medicina.

[76] Magalhaes R.S., Atala A., Atala A., Lanza R., Mikos A.G., Nerem R. (2019). Chapter 70—Regenerative Medicine for the Female Reproductive System. Principles of Regenerative Medicine.

[77] Sysak R., Bluska P., Stencl P., Klein M., Varga I. (2021). Agenesis of female internal reproductive organs, the Mayer- Rokitansky-Küster-Hauser syndrome. Bratisl. Lek. Listy.

[78] Campeanu R.A., Radu B.M., Cretoiu S.M., Banciu D.D., Banciu A., Cretoiu D., Popescu L.M. (2014). Near-infrared low-level laser stimulation of telocytes from human myometrium. Lasers Med. Sci..

[79] Popescu L.M., Gherghiceanu M., Manole C.G., Faussone-Pellegrini M.S. (2009). Cardiac renewing: Interstitial Cajal-like cells nurse cardiomyocyte progenitors in epicardial stem cell niches. J. Cell. Mol. Med..

[80] Popescu L.M., Manole E., Serboiu C.S., Manole C.G., Suciu L.C., Gherghiceanu M., Popescu B.O. (2011). Identification of telocytes in skeletal muscle interstitium: Implication for muscle regeneration. J. Cell. Mol. Med..

[81] Ceafalan L., Gherghiceanu M., Popescu L.M., Simionescu O. (2012). Telocytes in human skin--are they involved in skin regeneration?. J. Cell. Mol. Med..

[82] Popescu B.O., Gherghiceanu M., Kostin S., Ceafalan L., Popescu L.M. (2012). Telocytes in meninges and choroid plexus. Neurosci. Lett..

[83] Luesma M.J., Gherghiceanu M., Popescu L.M. (2013). Telocytes and stem cells in limbus and uvea of mouse eye. J. Cell. Mol. Med..

[84] Wang F., Song Y., Bei Y., Zhao Y., Xiao J., Yang C. (2014). Telocytes in liver regeneration: Possible roles. J. Cell. Mol. Med..

[85] Li L., Lin M., Li L., Wang R., Zhang C., Qi G., Xu M., Rong R., Zhu T. (2014). Renal telocytes contribute to the repair of ischemically injured renal tubules. J. Cell. Mol. Med..

[86] Grounds M.D. (2018). Obstacles and challenges for tissue engineering and regenerative medicine: Australian nuances. Clin. Exp. Pharmacol. Physiol..

[87] Varga I., Kyselovič J., Danišovič Ľ., Gálfiová P., Kachlík D., Polák Š., Klein M. (2019). Recently discovered interstitial cells termed telocytes: Distinguishing cell-biological and histological facts from fictions. Biologia.

